# Career Capital and Work Engagement in ICU Nurses: The Potential Mediating Role of Career Calling—A Cross‐Sectional Study

**DOI:** 10.1155/jonm/5555267

**Published:** 2026-07-30

**Authors:** Mengjiao Zhao, Feifei Dong, Xiaojing Guo, Yuchen Zhang, Ming Wei, Yubiao Gai

**Affiliations:** ^1^ Department of Critical Medicine, The Affiliated Hospital of Qingdao University, Qingdao 266000, Shandong, China, qdu.edu.cn; ^2^ Department of Critical Medicine, Head Nurse of the Department of Critical Care Medicine, The Affiliated Hospital of Qingdao University, Qingdao 266000, Shandong, China, qdu.edu.cn

**Keywords:** career calling, career capital, ICU, mediation effect, nurses, work engagement

## Abstract

**Aim:**

This research sought to explore the potential mediating function of career calling in the association between career capital and work engagement among intensive care unit (ICU) nurses, using the Job Demands–Resources (JD‐R) framework.

**Background:**

The work engagement of ICU nurses is considered important for nursing quality and patient recovery outcomes. Although previous studies have indicated close relationships among career capital, career calling, and work engagement, the underlying mechanisms remain obscure. The present research seeks to provide insights into this area.

**Design:**

A cross‐sectional design.

**Methods:**

The STROBE reporting guidelines were utilized. A total of 269 ICU nurses were recruited from a tertiary general hospital using convenience sampling. Data were collected using the Career Capital Scale, Calling Scale‐12 (CS‐12), and Utrecht Work Engagement Scale (UWES). Confirmatory factor analysis and common method bias assessment were conducted using SPSS AMOS 24.0. Descriptive statistics, Pearson correlation analysis, and multiple linear regression analysis were performed using SPSS 23.0. The significance of the mediating effect was tested using the PROCESS macro 4.1 plugin.

**Results:**

Both career capital and career calling were included in the regression model, accounting for 66.1% of the variance in work engagement. Career capital showed significant direct and indirect effects on work engagement, suggesting a partial mediating role of career calling in the relationship between career capital and work engagement.

**Conclusion:**

Higher levels of career capital among ICU nurses were associated with stronger intrinsic motivation, a greater sense of professional meaning and work value, and higher career calling. This pathway may be important for understanding work engagement.

**Implications for Nursing Management:**

Policymakers and managers may consider implementing ICU‐specific training (e.g., ACLS and ECMO), structured debriefings, peer support, and mentorship as potential strategies to support clinical competence, psychological safety, and career calling. These measures might be related to work engagement and nursing care quality, though causal inferences cannot be drawn from this cross‐sectional study.

**Summary**
• What does this study contribute to the wider global clinical community?
◦ The present research highlights the association between work engagement and nursing care quality.
◦ This investigation demonstrated that career capital and work engagement are significantly correlated in the ICU nursing population.
◦ This work established and confirmed a mediated framework that examines the relationship between career capital and work engagement and further illuminates the mediating function of career calling.

## 1. Introduction

The intensive care unit (ICU) is a medical unit dedicated to the centralized treatment of critically ill patients, characterized by a high‐intensity and high‐stress working environment. Consequently, ICU nurses are highly susceptible to professional burnout and turnover intention, creating challenges for sustaining strong work engagement [1]. During the care of critically ill patients, ICU nurses frequently encounter emergencies that demand heightened attention to patient safety and rapid response to clinical changes. This necessitates not only advanced clinical skills but also significant time investment [2]. Previously, researchers have predominantly paid attention to the negative emotional experiences of ICU nurses while overlooking the potential positive health outcomes for both patients and nurses under such demanding conditions. Following the growth of positive psychology, researchers have gradually turned their focus to the work engagement of nurses.

Work engagement denotes an enduring positive mindset that individuals exhibit, reflected in abundant vitality, dedication, and absorption. It stands as a crucial sign of workplace well‐being [3]. Currently, inadequate policies regarding nursing human resource allocation in China have resulted in a nurse‐to‐patient ratio below international standards, leading to chronic overwork and exacerbating work pressure [4]. Moreover, frequent night shifts and disruptions to circadian rhythms directly impair concentration, thereby reducing work engagement [5]. Evidence suggests that increased work engagement is linked to superior nursing quality, enhanced clinical competence, and facilitated recovery processes, including a reduction in patients’ inflammatory markers [6]. Therefore, investigating the factors associated with ICU nurses’ work engagement and improving their job satisfaction has become imperative.

In 2001, Demerouti et al. [7] proposed the Job Demands–Resources (JD‐R) model based on Lee and Ashforth’s [8] distinction between job demands and job resources. This model describes two concurrent psychological mechanisms. Excessive job demands trigger burnout through a health impairment process, whereas job resources enhance work engagement via a motivational process. From a positive psychology standpoint, the present research specifically concentrates on the motivational pathway within the JD‐R model. How job resources, as well as personal resources, could increase work engagement for ICU nurses is the central question addressed here.

Crucially, while the ICU environment is defined by extreme job demands, including heavy workload, emotional stress, and shift work, the JD‐R model indicates that resources become especially valuable in these high‐demand situations. This is because resources can lessen the harmful consequences of demands while fostering work engagement (Bakker and Demerouti, 2014). Within this framework, both career capital and career calling can be conceptualized as critical resources that may help ICU nurses cope with and mitigate the effects of these inherent occupational stressors.

Notably, career capital—an important concept for predicting individual and organizational behavior [9]—refers to the accumulation of various resources acquired through education and learning during one’s career development, encompassing both job resources and personal resources. It enables individuals to seize opportunities and overcome challenges, thereby facilitating long‐term development, enhancing professional competitiveness, and serving as a critical element for career success [10]. In the demanding ICU setting, career capital may provide nurses with the skills, knowledge, and networks needed to navigate high‐pressure situations more effectively, thereby conserving engagement energy. While previous research has emphasized the role of career capital in fields such as sociology [11], studies focusing on nurses’ career capital remain limited. Existing evidence suggests that the accumulation of career capital can significantly improve work engagement [12]. Therefore, understanding how to enhance ICU nurses’ career capital and strengthening their adaptability to professional roles represent pressing issues worthy of in‐depth investigation.

Career calling refers to a deep‐seated psychological drive through which individuals choose and commit to a profession based on intrinsic motivation and value alignment. Its core lies in transcending external conditions or material incentives, emphasizing the sense of meaning and purpose perceived in professional activities, as well as the willingness to contribute to collective well‐being and social welfare. It reflects an integration of personal life meaning and social value. As a positive personal resource, career calling may serve as a powerful motivational force that helps nurses reframe demanding situations as meaningful challenges rather than mere stressors, thereby sustaining their engagement. Research suggests that nurses who possess a stronger career calling tend to attach more importance to professional meaning and work values, to feel an elevated sense of duty, and to express greater satisfaction with their jobs [13]. Multiple studies have confirmed that career calling is associated with greater job satisfaction, less burnout, and fewer intentions to quit. This association thus holds practical significance for maintaining the stability of the nursing staff [14].

Existing research suggests that both career capital and career calling are significantly correlated with work engagement, and career calling is a significant predictor of work engagement. Although empirical evidence supports that career capital predicts work engagement positively, its underlying mechanism requires further exploration. According to the JD‐R model, both job resources and personal resources collectively influence work engagement through the motivational process. Career calling represents a favorable personal psychological resource, reflecting how strongly an individual feels passion and emotional devotion toward a certain field [15]. It is not only a manifestation of intrinsic motivation but also a crucial motivational force that significantly shapes an individual’s work attitudes and behavioral outcomes [16]. Hence, career calling might act as an intermediary variable connecting career capital with work engagement.

It is important to acknowledge that, in line with our focus on the motivational process, the present study does not empirically measure job demands. Consequently, our analysis addresses only the resource‐driven pathway of the JD‐R framework. While this approach allows for a focused examination of how career capital and career calling contribute to work engagement, it does not capture the full complexity of the JD‐R model, which posits dynamic interactions between demands and resources. Future research could adopt a more comprehensive modeling strategy, such as structural equation modeling (SEM), to simultaneously examine the health impairment and motivational processes, thereby providing a more holistic test of the JD‐R framework in the ICU context.

### 1.1. Aims and Hypotheses

**FIGURE 1:**
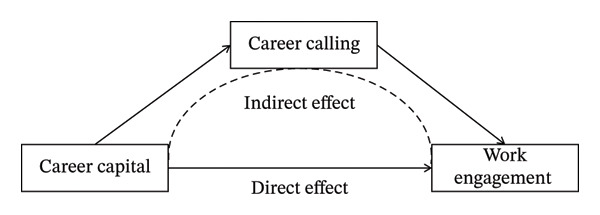
Theoretical model of this study.

The present work intends to test the mediation effect that career calling exerts on the association of career capital with work engagement. Based on review of the literature [12, 17] and the JD‐R model [18], three hypotheses were proposed: (1) ICU nurses’ work engagement is at a moderate level; (2) career capital is directly associated with work engagement; and (3) an indirect link exists from career capital to work engagement among ICU nurses, with career calling acting as the mediating factor. The theoretical model is illustrated in Figure 1.

## 2. Methods

### 2.1. Participants and Settings

This cross‐sectional study adhered to the Strengthening the Reporting of Observational Studies in Epidemiology (STROBE) guidelines. A convenience sampling method was employed to recruit 269 ICU nurses from a tertiary general hospital between April and June 2025. Data were collected through electronic questionnaires. The inclusion criteria were as follows: (1) possession of a valid nurse practitioner qualification certificate; (2) independently responsible for ICU nursing duties; and (3) engaged in clinical nursing work in the ICU for at least 3 months. The exclusion criteria included (1) nurses absent due to leave, further training, or rotation; (2) nursing interns or visiting nurses; and (3) ICU administrators not involved in clinical nursing. Participation in the study was voluntary, and each participant signed a written informed consent form.

According to Kendall’s principle for estimating sample sizes, the necessary number of participants is 5–10 times the count of variables, with 10–20 times the number of variables often recommended [19]. This study included 14 variables, resulting in an estimated sample size range of 70–140. Considering a potential nonresponse or invalid response rate of 10%–20%, the required minimum sample size was set at 77. Initially, 300 participants were recruited in total. After excluding questionnaires with excessively short completion times (< 3 min) or patterned responses, 269 valid questionnaires were included in the analysis. It should be noted that mediation analysis generally requires larger samples than basic regression. However, the achieved sample size of 269 exceeds the recommended minimum for testing indirect effects using PROCESS Model 4, which typically requires 100–200 participants for detecting medium effect sizes [20, 21]. Thus, our sample provides adequate statistical power for the mediation analysis.

### 2.2. Data Collection

Following approval from hospital administrators and head nurses, data were collected electronically using the Questionnaire Star platform. To minimize potential common method bias, several procedural remedies were implemented: (1) participants were assured of the anonymity and confidentiality of their responses; (2) the questionnaire used established scales with varied response formats; and (3) predictor and criterion variables were placed in separate sections of the questionnaire to reduce priming effects. The “Title and Description” section of the questionnaire contained the informed consent form for this study. Researchers clarified the aims and significance of the study to ICU nurses before they agreed to participate. An anonymous survey submission was initiated subsequent to obtaining informed consent. The questionnaire introduction included contact details of the research team to facilitate communication should participants have any inquiries. To ensure data quality, mandatory response settings, submission frequency restrictions, and submission controls were implemented. Submitted responses were reviewed by the research team via the Questionnaire Star backend. Data validation criteria included completion time and logical consistency of responses.

#### 2.2.1. Demographic Information Questionnaire

Drawing on a comprehensive review of the literature and input from experts, the research team created a self‐administered demographic questionnaire. It included items such as age, gender, marital status, having children, educational level, monthly income, ICU type, actual weekly working hours, average monthly night shift frequency, years of ICU work experience, and health status.

#### 2.2.2. Career Capital Scale

Chen [22] developed the scale within the framework of the intelligent career model and integrated its core dimensions of human capital and social capital. This scale contains 13 items distributed across three dimensions: knowing‐why, knowing‐how, and knowing‐whom. Each item uses a 5‐point Likert scale where 1 indicates “*strongly disagree*” and 5 indicates “*strongly agree*.” Aggregate scores fall in the range of 13–65, and elevated scores correspond to more ample career capital. In the ICU nursing context, knowing‐why refers to nurses’ professional mission (e.g., finding meaning in saving critically ill patients); knowing‐how includes technical skills (e.g., ECMO, CRRT, and ventilator management); and knowing‐whom encompasses collaborative relationships (e.g., teamwork with physicians and peer support). The instrument has been proven to have both strong reliability and good validity. In this study, the overall Cronbach’s *α* coefficient was 0.980, with subscale coefficients ranging from 0.954 to 0.966. The scale has previously been applied among emergency nurses by Ye et al. [12].

#### 2.2.3. Calling Scale‐12 (CS‐12)

The Career Calling Scale, originally developed by Dobrow and Tosti‐Kharas [23], was translated into Chinese by Pei and Zhao [24] and later revised by Shen and Qian [13]. This unidimensional scale consists of 12 items rated on a 5‐point Likert scale ranging from 1 (“strongly disagree”) to 5 (“strongly agree”). Total scores range from 12 to 60, with mean scores < 3 classified as low calling and ≥ 3 classified as high calling. Higher scores indicate stronger value alignment with one’s profession and more pronounced intrinsic work motivation. Previous studies have demonstrated the scale’s applicability among Chinese nurses [25]. In this study, the Cronbach’s *α* coefficient was 0.968.

#### 2.2.4. Utrecht Work Engagement Scale (UWES)

Based on the three‐dimensional theory of work engagement proposed by Schaufeli et al. [26], the Chinese version was translated and adapted by Zhang and Gan [27]. The scale emphasizes three core dimensions: vigor, dedication, and absorption, comprising 17 items in total. Each item is rated on a 7‐point Likert scale from 0 (“*never*”) to 6 (“*always*”), with total scores ranging from 0 to 102. Higher scores indicate greater work engagement. The scale has shown strong applicability among knowledge workers and is widely used in nursing research [28]. In this study, the overall Cronbach’s *α* coefficient was 0.975, with subscale coefficients ranging from 0.936 to 0.956.

### 2.3. Data Analysis

The psychometric features of the three instruments were further assessed by means of confirmatory factor analysis (CFA) implemented in SPSS AMOS 24.0 (IBM Corp, Armonk, New York). Composite reliability (CR) and average variance extracted (AVE) were calculated to assess convergent validity, with CR > 0.70 and AVE > 0.50, indicating acceptable convergent validity.

To assess the potential threat of common method bias, we conducted Harman’s single‐factor test and a series of CFA model comparisons, including an unmeasured latent method construct (ULMC) analysis [29, 30].

Data processing and statistical analysis were performed using SPSS 23.0 (IBM Corp, Armonk, New York). Continuous variables were expressed as means and SDs, while categorical variables were described using frequencies and percentages. Independent samples *t*‐tests or one‐way analysis of variance (ANOVA) was employed to examine differences in work engagement across demographic characteristics. Pearson correlation analysis was used to assess the relationships among work engagement, career capital, and career calling.

Covariates were selected based on their bivariate associations with work engagement (Table 1). Among all sociodemographic variables, only health status showed a statistically significant association with work engagement (*p* < 0.001). Therefore, considering the sample size (*N* = 269) and the number of predictors in the full model, health status was entered as the sole control variable in Model 1 to preserve statistical power and avoid overfitting. Other variables did not exhibit significant bivariate associations (*p* > 0.05) and were thus not included as covariates. A hierarchical multiple regression analysis (forced‐entry method) was then conducted to examine the unique contributions of the variables. In Model 1, health status was entered as a control variable. In Model 2, career capital was added. In Model 3, career calling was incorporated. Within each model, all variables were entered simultaneously, and no automatic stepwise selection procedure was applied.

**TABLE 1:** Sociodemographic of ICU nurses (*N* = 269).

Variables	M ± SD/*N* (%)	Total score of work engagement (SD)	*t* or *F*	*p*
Age	32.13 ± 5.478	72.36 (20.79)	0.825	0.708
Gender			1.951	0.164
Male	79 (29.4)	75.10 (20.31)		
Female	190 (70.6)	71.22 (20.93)		
Marriage status			1.493	0.223
Married	187 (69.5)	73.39 (20.51)		
Single	82 (30.5)	70.02 (21.36)		
Have children			3.490	0.063
Yes	166 (61.7)	74.22 (20.68)		
No	103 (38.3)	69.37 (20.71)		
Educational level			0.031	0.969
College degree or below	14 (5.2)	73.71 (20.66)		
University degree	242 (90)	72.29 (21.08)		
Master’s degree or above	13 (4.8)	72.23 (16.11)		
Monthly income (US dollars)			2.214	0.111
Below 680	18 (6.7)	78.33 (26.18)		
680–1360	178 (66.2)	73.34 (20.51)		
Above 1360	73 (27.1)	68.51 (19.66)		
ICU type			0.007	0.993
Emergency ICU	8 (3)	72.25 (18.46)		
Comprehensive ICU	237 (88.1)	72.32 (20.05)		
Specialized ICU	24 (8.9)	72.83 (28.37)		
Actual weekly working hours (h)			2.402	0.057
< 40	53 (19.7)	78.45 (19.40)		
> 40	216 (80.3)	70.87 (20.89)		
Average monthly night‐shift frequency (units)			0.412	0.745
0	16 (5.9)	76.63 (17.67)		
1–3	17 (6.3)	75.53 (23.49)		
4–6	67 (24.9)	71.48 (20.17)		
7–10	169 (62.8)	71.99 (21.11)		
Years of ICU work experience			1.168	0.325
< 1	20 (7.4)	75.70 (17.73)		
1–3	49 (18.2)	72.14 (21.59)		
4–7	67 (24.9)	74.01 (21.45)		
8–10	43 (16)	66.42 (19.92)		
> 10	90 (33.5)	73.34 (20.77)		
Health status			5.714	< 0.001^a^
Health	118 (43.9)	77.79 (19.52)		
Good	89 (33.1)	69.99 (19.12)		
General	60 (22.3)	65.65 (23.25)		
Poor	2 (0.7)	59.00 (11.31)		

^a^ *p* < 0.001.

The mediating effect of career calling on the relationship between career capital and work engagement was tested using Model 4 of the PROCESS macro 4.1 plugin (Andrew F. Hayes, Calgary, Canada). A bootstrap resampling method with 5000 replicates was applied [31]. The presence of a significant mediation effect was determined by whether the bias‐corrected 95% confidence interval (CI) for the indirect effect (*a* ∗ *b*) did not contain zero.

## 3. Results

### 3.1. Reliability and Validity of the Measurement Scales

As shown in Table 2, all dimensions demonstrated satisfactory internal consistency, with Cronbach’s *α* coefficients ranging from 0.936 to 0.968. CR values ranged from 0.939 to 0.969, and AVE values ranged from 0.719 to 0.879, confirming adequate convergent validity. The results indicated that the scales possessed satisfactory reliability and validity for use in this study.

**TABLE 2:** Reliability and validity of the measurement scales.

Scales	Number of items	Cronbach’s *α*	CR	AVE
Career capital	13	0.980	—	—
Know‐why	4	0.954	0.955	0.843
Know‐how	5	0.963	0.965	0.846
Know‐who	4	0.966	0.967	0.879
Career calling	12	0.968	0.969	0.726
Work engagement	17	0.975	—	—
Vitality	6	0.956	0.958	0.794
Dedication	5	0.936	0.944	0.777
Concentration	6	0.937	0.939	0.719

Abbreviations: AVE, average variance extracted; CR, composite reliability.

### 3.2. Common Method Bias Assessment

Harman’s single‐factor test showed that the first unrotated factor explained 60.546% of the total variance, slightly exceeding the conventional 50% threshold.

CFA model comparisons were then performed. The single‐factor model, in which all items were forced to load onto one latent factor, demonstrated poor fit (*χ* ^2^/*d* *f* = 9.259, CFI = 0.583, IFI = 0.584, TLI = 0.562, RMSEA = 0.176). In contrast, the hypothesized three‐factor model exhibited excellent fit (*χ* ^2^/*d* *f* = 2.677, CFI = 0.990, IFI = 0.990, TLI = 0.983, RMSEA = 0.079).

ULMC analysis was subsequently conducted by adding a common latent factor to the three‐factor model, with all items loading on both their respective trait factors and the method factor. The ULMC model showed minimal improvement over the baseline three‐factor model, with all Δ values below the recommended thresholds (ΔCFI = −0.004, ΔIFI = −0.004, ΔTLI = 0.001, ΔRMSEA = −0.004). The ULMC model also maintained excellent fit (CFI = 0.994, IFI = 0.994, TLI = 0.982, RMSEA = 0.083).

Taken together, although Harman’s single‐factor test indicated a potential concern, the more rigorous ULMC analysis confirmed that common method bias does not substantially affect the substantive conclusions of this study. The complete model fit comparisons are presented in Table 3.

**TABLE 3:** Common method bias assessment: Model fit comparisons.

Model	CMIN/DF	RMSEA	CFI	IFI	TLI
Baseline three‐factor model	2.677	0.079	0.990	0.990	0.983
Single‐factor model	9.259	0.176	0.583	0.584	0.562
Common method factor model	2.840	0.083	0.994	0.994	0.982
Change in model fit (ULMC vs. Baseline)		ΔRMSEA	ΔCFI	ΔIFI	ΔTLI
		−0.004	−0.004	−0.004	0.001

*Note:* Δ represents change from the baseline three‐factor model to the ULMC model. Acceptance criteria: ΔRMSEA < 0.05 and ΔCFI/ΔIFI/ΔTLI < 0.1 [29, 32]. CMIN/DF, chi‐square to degrees of freedom ratio; RMSEA, root mean square error of approximation.

Abbreviations: CFI, comparative fit index; IFI, incremental fit index; TLI, Tucker–Lewis index; ULMC, unmeasured latent method construct.

### 3.3. Descriptive Statistics of Variables

Table 4 presents the means, SDs, and range of scores for the dimensions of career capital, career calling, and work engagement. The mean work engagement score among ICU nurses was 72.36 (SD = 20.79), indicating a moderately high level of engagement.

**TABLE 4:** Scores for each dimension of the scales.

	Range of scores	Means	SDs
Career capital	13∼65	56.66	8.77
Know‐why	4∼20	17.33	2.85
Know‐how	5∼25	22.04	3.32
Know‐who	4∼20	17.28	3.02
Career calling	12∼60	48.23	10.05
Work engagement	0∼102	72.36	20.79
Vitality	0∼36	24.79	8.23
Dedication	0∼30	21.55	6.24
Concentration	0∼36	26.02	7.35

### 3.4. Demographic Characteristics

The final sample consisted of 269 ICU nurses, with an average age of 32.13 years (SD = 5.48). Among participants aged 22–49 years, statistically significant differences in work engagement were observed across different health status groups (*p* < 0.001). Specifically, nurses with poor self‐rated health exhibited reduced work engagement. Table 1 presents a summary of the remaining demographic variables.

### 3.5. Correlation Analysis of Career Capital, Career Calling, and Work Engagement

As presented in Table 5, results of the correlation analysis indicated meaningful positive links between all variables examined in this study. Career capital was positively correlated with career calling (*r* = 0.668, *p* < 0.01) and work engagement (*r* = 0.603, *p* < 0.01). Similarly, career calling demonstrated a strong positive correlation with work engagement (*r* = 0.808, *p* < 0.01).

**TABLE 5:** Correlation of career capital, career calling, and work engagement.

	Career capital	Career calling	Work engagement
Career capital	1		
Career calling	0.668^∗∗^	1	
Work engagement	0.603^∗∗^	0.808^∗∗^	1

^∗∗^At the 0.01 level (two‐tailed), the correlation is significant.

### 3.6. The Mediating Effect of Career Calling Between Career Capital and Work Engagement

A multiple linear regression model (forced‐entry method) was constructed using the hierarchical method to identify significant predictors of work engagement. In the hierarchical regression, health status was entered in Model 1 as a control variable. Career capital was entered in Model 2, and career calling was entered in Model 3. As all variables were forced into the model at their respective steps, each variable remained in the model regardless of its significance level.

As shown in Table 6, health status, while showing a significant bivariate correlation with work engagement, did not show a significant unique predictive effect in the final regression model after the inclusion of career capital and career calling (*p* > 0.05), indicating that its predictive power was attenuated when accounting for these psychological resource variables. Consistent with the forced‐entry hierarchical approach, health status was retained in the model as specified; only its individual contribution was nonsignificant. Model 2 (*F* = 78.167, *p* < 0.05, *R* ^2^ = 0.370) explained 37.0% of the variance, with career capital accounting for an additional 31.1% (Δ*R* ^2^ = 0.311) after controlling for health status. In Model 3 (*F* = 172.090, *p* < 0.001, *R* ^2^ = 0.661), career calling entered the model, and together with career capital, the model accounted for 66.1% of the variance in work engagement. Career calling emerged as the explanatory variable, contributing an additional 29.1% of the explained variance.

**TABLE 6:** Hierarchical regression analysis results.

Variables	*B*	SE	*β*	*t*	*p*	95% CI	*R* ^2^	Δ*R* ^2^	*F*
Model 1							0.059	0.059	16.826
Health status	−6.260	1.526	−0.243	−4.102	< 0.001	(−9.265, −3.255)			
Model 2							0.370	0.311	78.167
Health status	−2.252	1.299	−0.088	−1.733	0.084	(−4.809, 0.306)			
Career capital	1.373	0.120	0.579	11.458	< 0.001	(1.137, 1.609)			
Model 3							0.661	0.291	172.090
Health status	−0.306	0.964	−0.012	−0.317	0.751	(−2.203, 1.592)			
Career capital	0.263	0.115	0.111	2.289	< 0.05	(0.037, 0.489)			
Career calling	1.512	0.100	0.731	15.069	< 0.001	(1.315, 1.710)			

Abbreviation: CI, confidence intervals.

**FIGURE 2:**
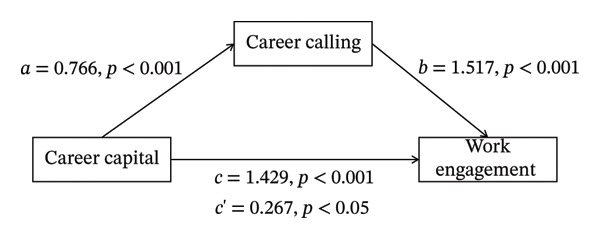
The mediating effect of career calling between career capital and work engagement. *Note:* path coefficients are unstandardized regression coefficients (*B*).

Further testing of career calling’s mediation effect was conducted with the PROCESS macro. Figure 2 shows that career capital exerted a significant total effect on work engagement (*c* path: *B* = 1.429; *p* < 0.001; 95% CI, 1.201–1.657). After adjusting for covariates, the direct effect of career capital on career calling (*a* path: *B* = 0.766; *p* < 0.001; 95% CI, 0.663–0.869) and the direct effect of career calling on work engagement (*b* path: *B* = 1.517; *p* < 0.001; 95% CI, 1.321–1.712) remained significant. The direct effect of career capital on work engagement stayed significant after additional adjustments were made for career calling and other covariates (*c*′ path: *B* = 0.267; *p* < 0.05; 95% CI, 0.043–0.491). Additionally, career capital exerted a significant indirect effect on work engagement through career calling (*a* ∗ *b* = 1.162; 95% bias‐corrected bootstrap CI, 0.956–1.384), indicating that career calling partially mediates the relationship between career capital and work engagement. Other relevant data are presented in Table 7.

**TABLE 7:** The result of mediation analysis (*N* = 269).

Path	Coefficient	SE	*t*	*p*	95% CI	
					LLCI	ULCI
Total effect						
Career capital ⟶ work engagement (*c*)	1.429	0.116	12.336	< 0.001	1.201	1.657
Direct effect						
Career capital ⟶ career calling (*a*)	0.766	0.052	14.672	< 0.001	0.663	0.869
Career calling ⟶ work engagement (*b*)	1.517	0.099	15.275	< 0.001	1.321	1.712
Career capital ⟶ work engagement (*c*′)	0.267	0.114	2.348	< 0.05	0.043	0.491
Indirect effect						
Career capital ⟶ career calling ⟶ work engagement (*a* ∗ *b*)	1.162	0.109	—	—	0.956	1.384

Abbreviations: CI, confidence interval; LLCI, lower‐limit confidence interval; SE, standard error; ULCI, upper‐limit confidence interval.

## 4. Discussion

The primary objective of this study was to investigate the direct relationship and underlying pathways between career capital and work engagement among ICU nurses based on the JD‐R model. Findings indicated a significant relationship between career capital and work engagement, with career calling playing a partial mediating role in this relationship.

### 4.1. Current Status of Work Engagement Among ICU Nurses

The mean total score on the UWES was 72.36 (SD = 20.79), reflecting work engagement that is moderately high in comparison with the scale’s theoretical median of 51. The current outcome aligns with the data documented by Liu et al. [2], but is notably higher than the outcomes observed among emergency department nurses [33] and general ward nurses [34]. Several factors may explain this phenomenon: the investigated hospital had well‐established policies and an effectively implemented flexible scheduling system; furthermore, the ICU environment, while dealing with critically ill patients, offers a relatively controlled setting with lower nurse‐to‐patient ratios and demands for highly specialized knowledge and skills. The combination of professional autonomy, opportunities for specialized practice, strong team support, and timely feedback aligns well with the core motivational elements for knowledge workers. These circumstances allow nurses to preserve strong enthusiasm and engagement despite significant physical and psychological pressures. High work engagement is significantly associated with lower burnout and turnover intention among nurses [35]. Nursing managers should fully acknowledge the value of ICU nurses’ roles, which could be associated with high‐quality development in critical care nursing.

It is noteworthy that an interesting finding from the regression analysis warrants further discussion within the context of work engagement levels. Health status did not show a significant unique predictive effect in the final multiple regression model, contrasting with prior research conclusions [36]. This suggests that, after career capital and career calling are introduced, the predictive power of health status on work engagement may be largely explained or superseded by more potent psychological resources. The motivational pathway within the JD‐R model offers a way to understand this phenomenon. While poor health status may be considered a personal demand that may impair energy and focus, the presence of job resources appears to activate a powerful motivational pathway that overshadows the impact of physical condition. In other words, for ICU nurses who possess a rich reservoir of skills, knowledge, and social networks, and who view their work as a meaningful calling, their psychological drive and work engagement may be less dependent on their own health status. This aligns with the resource buffering hypothesis of the JD‐R framework. It implies that high‐quality resources are capable of mitigating the adverse effects of job demands, allowing nurses to sustain high engagement levels despite high‐pressure environments. From a managerial perspective, this finding is encouraging: even when nurses face physical challenges in the demanding ICU environment, investing in their career capital and fostering their sense of calling can serve as powerful levers to sustain high levels of work engagement.

### 4.2. Direct Effect of Career Capital on Work Engagement Among ICU Nurses

The results indicate a positive link between career capital and work engagement, confirming Hypothesis 2. The enhancement of job resources together with personal resources has been shown in previous studies to expand organizational and external variables, thereby generating positive effects and increasing work engagement [37]. This result is broadly in line with earlier research [12]. According to the motivational pathway of the JD‐R model, job resources serve as a core variable influencing work engagement [38]. Career capital among ICU nurses may help fulfill intrinsic psychological needs by cultivating feelings of efficacy, support, and value, and this capital also correlates with strong internal motivation. Consequently, this factor may relate to whether nurses can maintain the three core facets of high work engagement (vitality, dedication, and absorption) despite working in highly challenging conditions.

Compared to nurses with lower career capital, those with higher career capital tend to more effectively leverage their knowledge, skills, teamwork, and psychological resilience, which may be related to responding to job demands more rapidly. During critical situations such as resuscitation events, they can quickly comprehend and execute instructions, sustaining high work engagement and preventing burnout. Based on these findings, nursing managers may consider developing policies at both departmental and hospital levels that could support continuous learning, such as protecting time for professional development and designing compensation systems that reflect the value of career capital appropriately. A unit culture that emphasizes learning and collective growth may be associated with a virtuous cycle of career capital accumulation, clearer career development pathways, and higher work engagement.

### 4.3. Partial Mediating Effect of Career Calling Between Career Capital and Work Engagement

Career calling represents a passionate sense of mission or responsibility, reflecting a transcendent perception of purpose beyond the self [39]. As an intrinsic motivation, it is associated with an individual’s engagement and satisfaction with work [25]. Based on the JD‐R model, our mediation analysis further revealed that career calling among ICU nurses was positively correlated with both career capital and work engagement. Moreover, career calling partially accounted for the indirect effect of career capital on work engagement, confirming Hypothesis 3. Career capital is associated with work engagement, and this association may be linked to ICU nurses’ career calling, which is consistent with prior research [40].

Job resources are positively associated with career calling. Nurses with access to greater job resources tend to exhibit stronger professional identity, maintain positive attitudes toward nursing, and demonstrate heightened enthusiasm for clinical work, and such characteristics often correspond to a stronger sense of career calling [41]. In turn, career calling links to how nurses adapt psychologically on the job. It may be related to work commitment by fostering a sense that one’s profession is an indispensable part of one’s identity, which may encourage nurses to engage wholeheartedly with positive emotions and sustained enthusiasm, thereby enhancing their work engagement. As indicated by Min et al. [42], career calling is essentially driven by psychological goals and serves as a motivational force that promotes favorable work attitudes. It may help nurses develop a deeper affection for their work; this affection is correlated with positive workplace behaviors, constructive professional values, and higher work engagement.

This finding indicates that career calling only partially mediates the link between career capital and work engagement, implying that other factors may also play a role. From the JD‐R perspective, several additional mediating factors may explain the remaining variance. First, self‐efficacy, a core personal resource, may serve as an independent pathway; career capital is associated with nurses’ confidence in handling critical situations such as resuscitation, and this heightened self‐efficacy may be related to work engagement by fulfilling the need for competence [43]. Second, psychological safety, a critical job resource, may act as a mediator; the relational networks embedded in career capital enable nurses to collaborate effectively with physicians and colleagues, creating a trusting environment where proactive engagement can flourish [44]. Third, job crafting, an active behavioral process in the extended JD‐R model, may represent a distinct pathway through which career capital is associated with work engagement. This may occur via proactive behaviors such as seeking learning opportunities, optimizing workflows, and building supportive relationships, which have been linked to resource gain and demand optimization [12].

These findings suggest that nursing administrators may consider developing ICU nurses’ career capital through skill development, team support, resilience cultivation, and professional recognition. This approach could relate to a greater sense of mission and value (i.e., career calling). This sense of meaning may in turn be correlated with nurses’ proactive and resilient engagement in their work, which has been linked to mutual growth for both individuals and the organization in high‐pressure environments. Subsequent studies might explore these potential pathways in greater depth to develop a more comprehensive model of the factors associated with work engagement among ICU nurses.

## 5. Implications for Nursing Management

Nursing management can draw meaningful lessons from this study, whose implications are centered on four critical aspects: developing career capital, promoting psychological safety, enhancing career calling, and increasing public acknowledgment. First, nursing administrators ought to give priority to building career capital in ICU nurses through targeted professional development initiatives. The implementation of ICU‐specific training programs, including continuous renal replacement therapy (CRRT), advanced cardiovascular life support (ACLS), extracorporeal membrane oxygenation (ECMO) management, ventilator management, and high‐fidelity simulation‐based training, together with clearly defined career advancement pathways, may be associated with higher clinical competence. As an illustrative example, hospitals might consider establishing a tiered “Credentialed ICU Nurse” pathway requiring completion of core competency modules with skills verification, which could be linked to career progression and compensation adjustments. In turn, this may be associated with nurses’ confidence in responding to complex clinical challenges, as well as higher self‐efficacy and proactive work engagement. Second, fostering a psychologically safe environment tailored to the ICU context is essential. Given the inherently high‐stress nature of intensive care work, nursing managers should establish structured debriefing sessions following critical incidents such as cardiac arrests. For instance, facilitator‐led debriefing sessions conducted shortly after critical events, which follow a structured format that reviews clinical actions and processes emotional responses, could be implemented as one practical approach. Complemented by daily safety huddles and peer support programs, such practices can mitigate burnout and reinforce psychological safety, ultimately providing nurses with the emotional and instrumental support needed to fully engage in their work. Third, since career calling is tied to career capital and work engagement and serves as a mediator between them, targeted interventions to strengthen career calling may effectively enhance work engagement. Nursing managers can support career calling by granting experienced nurses greater professional autonomy—for example, by involving them in leading complex care teams, serving as preceptors for novice nurses, and participating in ICU quality improvement committees. Additionally, structured mentorship programs that pair new nurses with seasoned preceptors can further solidify professional identity. Such approaches, including regular mentor–mentee meetings and collaborative case discussions, can be adapted based on institutional resources. These approaches may be associated with intrinsic motivation, a sense of responsibility and value, and career calling among nurses. Finally, government administrators in China may consider launching media campaigns that highlight the life‐saving contributions of ICU nurses and instituting policy‐level recognition, such as specialized awards or an “ICU Nurse Day.” For example, establishing a national recognition program (e.g., annual excellence awards or a designated awareness week) could be one potential avenue that may be associated with greater public awareness of critical care nursing. Such initiatives were correlated with the social standing of the nursing profession and nurses’ career calling. These measures also showed correlations with work engagement and nursing care quality. It should be noted, however, that these practical examples are offered as hypothesis‐generating possibilities for future intervention research, rather than as evidence‐based policy prescriptions directly derived from our cross‐sectional data.

## 6. Limitations

New knowledge about factors affecting work engagement for ICU nurses emerges from this study; nevertheless, certain limitations require mention. First, causal interpretations of variable relationships are restricted due to the cross‐sectional approach. Subsequent studies might employ longitudinal methods to strengthen the validation of these relationships. Second, employing self‐reported surveys to collect data could introduce bias into the information obtained. Specifically, Harman’s single‐factor test indicated that the first unrotated factor explained 60.55% of the total variance, slightly exceeding the conventional 50% threshold [29]. However, given the recognized limitations of Harman’s test as a relatively conservative diagnostic tool [45, 46], we further employed the ULMC approach—a more rigorous method for detecting common method bias. The ULMC analysis showed that after controlling for a common method factor, model fit improved only minimally. This suggests that while some degree of common method variance may exist, its impact on the substantive conclusions is limited. Nevertheless, future studies could combine self‐evaluations with assessments from others (e.g., head nurses or managers) or adopt longitudinal designs to further mitigate this bias. Third, the generalizability of our findings is constrained by the sampling method. Data were collected using a convenience sample of ICU nurses recruited from one tertiary hospital in a single Chinese province. This single‐center design constrains the generalizability of our findings to nurses at other institutions, particularly those at different levels (e.g., secondary or primary hospitals) or in different geographical regions with varying work cultures and resources. To make conclusions more representative and externally valid, forthcoming studies should prioritize multicenter studies involving a broader range of hospitals across diverse regions. Finally, the exceptionally high Cronbach’s *α* coefficients (all > 0.93) warrant careful consideration. While these values indicate strong internal consistency, they may also suggest potential item redundancy. Further analysis showed that CR values ranged from 0.939 to 0.969 and AVE values from 0.719 to 0.879, confirming convergent validity while also reflecting substantial item overlap. Therefore, although the scales used in this study are well‐validated instruments, future research could consider employing shorter, validated versions of these scales to reduce respondent burden, particularly given the severe time constraints faced by ICU nurses, without compromising measurement quality.

## 7. Conclusions

To summarize, the present research revealed that ICU nurses demonstrated a moderately high degree of work engagement. Career capital shows a positive connection to work engagement, and the relationship between the two is partially mediated by career calling. These findings extend the JD‐R theory by suggesting a potential pathway through which job resources may be related to work engagement. Viewing the topic through positive psychology, the present study illuminates the relevant pathways by which personal and job resources influence work engagement among ICU nurses caring for critically ill patients, extending the application of the JD‐R model in this population and clarifying the motivational mechanism of career capital. Nursing administrators may consider approaches that may be associated with work engagement among their staff: empowering human capital by providing systematic training and career development support; strengthening social capital by fostering teamwork and a supportive work environment; investing in psychological capital, which may be related to resilience and self‐efficacy; and supporting cultural capital by reinforcing professional identity and organizational culture. These measures may help translate resources into intrinsic motivation, reduce workplace obstacles, and be associated with nurses’ sense of fulfillment and efficacy. Such approaches may be associated with nurses’ perception of the meaning and value of their profession, as well as with higher work engagement.

## Author Contributions

Yubiao Gai: conceptualization, writing–review and editing, supervision, and project administration. Mengjiao Zhao: conceptualization, methodology, formal analysis, investigation, data curation, writing–original draft, and visualization. Feifei Dong: methodology, formal analysis, investigation, data curation, and writing–original draft. Xiaojing Guo: methodology, formal analysis, and investigation. Yuchen Zhang: methodology, formal analysis, and investigation. Ming Wei: writing–review and editing, supervision, and project administration.

## Funding

This research received no specific grant from any funding agency in the public, commercial, or not‐for‐profit sectors.

## Ethics Statement

This study obtained approval from the ethics committee of the Affiliated Hospital of Qingdao University (No: QYFYWZLL 30523). Participants volunteered anonymously and were informed of the research purpose and procedures before participation. Researchers guaranteed participants the absolute privacy of their data and confirmed that their participation would not result in any adverse effects.

## Conflicts of Interest

The authors declare no conflicts of interest.

## Data Availability

The data that support the findings of this study are available on request from the corresponding authors. The data are not publicly available due to privacy or ethical restrictions.

## References

[1] Zhang C. Q. , Li X. , He L. et al., Critical Care, Critical Gaps: Assessment of Burnout and Behavioral Profiles of ICU Healthcare Workers in China-A Multicenter Cross-Sectional Study, Frontiers in Public Health. (2025) 13, 10.3389/fpubh.2025.1617081. PMC12240766 40642244

[2] Liu Q. , Zhao L. , Guo X. , Zhang Y. , Xin C. , and Gai Y. , Leisure Crafting and Work Engagement Among Chinese ICU Nurses: The Multiple Mediation Effect of Recovery Experience and Humanistic Care Ability, International Nursing Review. (2024) 71, no. 4, 776–785, 10.1111/inr.12914. 38041443

[3] Wee K. Z. and Lai A. Y. , Work Engagement and Patient Quality of Care: A Meta-Analysis and Systematic Review, Medical Care Research and Review. (2022) 79, no. 3, 345–358, 10.1177/10775587211030388. 34238065

[4] National Health Commission of the People’s Republic of China , National Nursing Career Development Plan (2021-2025), 2022, https://www.nhc.gov.cn/yzygj/s7653pd/202205/441f75ad347b4ed68a7d2f2972f78e67.shtml, [EB/OL]. (2022-04-29)[2022-07-12] (In Chinese).

[5] Zhao L. , Li C. , Yu H. et al., The Mediating Effect of Organizational Justice on the Relationship Between Person-Organization Fit and Work Engagement Among ICU Nurses, Japan Journal of Nursing Science. (2025) 40, no. 4, 62–65, (In Chinese) 10.3870/j.issn.1001-4152.2025.04.062.

[6] Chen H. X. , Liu W. , Liu B. et al., Influence of Work Engagement and Self-Efficacy of Nurses on Clinical Practice Ability in Burn Intensive Care Unit, Chinese Journal of Burns. (2023) 39, no. 8, 779–786, (In Chinese) 10.3760/cma.j.cn501225-20220905-00379. PMC11630266 37805790

[7] Demerouti E. , Bakker A. B. , Nachreiner F. , and Schaufeli W. B. , The Job Demands-Resources Model of Burnout, Journal of Applied Psychology. (2001) 86, no. 3, 499–512, 10.1037/0021-9010.86.3.499. 11419809

[8] Lee R. T. and Ashforth B. E. , A Meta-Analytic Examination of the Correlates of the Three Dimensions of Job Burnout, Journal of Applied Psychology. (1996) 81, no. 2, 123–133, 10.1037/0021-9010.81.2.123. 8603909

[9] Chen C. S. , Career Capital: Concept Connotation and Scale Improvement, Science Technology and Industry. (2018) 18, no. 4, 87–93, (In Chinese) 10.3969/j.issn.1671-1807.2018.04.015.

[10] Defillippi R. J. and Arthur M. B. , The Boundaryless Career: A Competency‐Based Perspective, Journal of Organizational Behavior. (2010) 15, no. 4, 307–324, 10.1002/job.4030150403.

[11] Rajala A. , The Relationship Between Career Capital and Career Success Among Finnish Knowledge Workers, Baltic Journal of Management. (2020) 15, no. 5, 687–705, 10.1108/BJM-10-2019-0357.

[12] Ye W. X. , Jin J. F. , Lan M. J. , and Zhang Y. P. , The Mediating Effect of Job Crafting Between Career Capital and Work Engagement Among Emergency Specialist Nurses, Chinese Journal of Nursing. (2024) 59, no. 23, 2841–2846, (In Chinese) 10.3761/j.issn.0254-1769.2024.23.004.

[13] Shen M. and Qian Z. G. , Career Calling and Its Influencing Factors Amongst Oncology Nurses in Anhui Province, Chinese Journal of General Practice. (2022) 20, no. 4, 708–712, (In Chinese) 10.16766/j.cnki.issn.1674-4152.002436.

[14] Zhao X. , Wu K. , Sun B. , and Li W. , Teacher Career Calling Reduces Burnout: The Mediation Effects of Work Engagement and Psychological Capital, Frontiers in Psychology. (2022) 13, 10.3389/fpsyg.2022.988467. PMC9671212 36405153

[15] Zhu M. , Qin L. L. , He C. Y. et al., Chain Mediating Effect of Perceived Social Support and Thriving at Work Between Nurses’ Job Crafting and Career Calling, The Journal of Nursing Administration. (2025) 25, no. 4, 282–286, (In Chinese) 10.3969/j.issn.1671-315x.2025.04.002.

[16] Zhou X. , Wang Y. , Xu Y. , Zhu H. , and Huang X. , The Role of Traditional Culture and Career Calling in Medical Staff Job Performance: An Examination Using Structural Equation Modeling, BMC Health Services Research. (2025) 25, no. 1, 10.1186/s12913-025-13290-8. PMC12341146 40797319

[17] Xi Y. , Zhou L. , and Guo D. , The Power of Calmness in Times of the COVID-19 Pandemic: The Different Roles of Peace of Mind and Career Calling in Enhancing Work Engagement-A Mediation Analysis Based on Social Support, Frontiers in Psychology. (2023) 14, 10.3389/fpsyg.2023.1104430. PMC10034166 36968745

[18] Demerouti E. , Job Demands: Resources Theory, 2014, Wellbeing.

[19] Preacher K. J. and Kelley K. , Effect Size Measures for Mediation Models: Quantitative Strategies for Communicating Indirect Effects, Psychological Methods. (2011) 16, no. 2, 93–115, 10.1037/a0022658. 21500915

[20] Fritz M. S. and Mackinnon D. P. , Required Sample Size to Detect the Mediated Effect, Psychological Science. (2007) 18, no. 3, 233–239, 10.1111/j.1467-9280.2007.01882.x. 17444920 PMC2843527

[21] Preacher K. J. and Hayes A. F. , Asymptotic and Resampling Strategies for Assessing and Comparing Indirect Effects in Multiple Mediator Models, Behavior Research Methods. (2008) 40, no. 3, 879–891, 10.3758/brm.40.3.879. 18697684

[22] Chen C. S. , Career Capital, Role-Identity Crafting and Dual Effects of Knowledge Workers’ Job-Hopping, 2018, Huaqiao University, https://kns.cnki.net/kcms2/article/abstract?v=52O9CKbg8L7Iqq9I2QWoWxwZrzMotu1y_2h0woh1HsEnJh-6Sn3xaSR4Rqz7G_DmeYTjHtVbzqXxqCG7Hcj2huZ-LhE58kBh9uuZJvTZKad9GdF1h8gpeMgbFtMVjj6CrHgXSgx-hr_j_FssAj0fA1HGNLMkqwp9apyBGroN7OstdeH2lePpU-sj_stTOubrzp0oWvDo9Rw=%26uniplatfo, Doctoral Dissertation.

[23] Dobrow S. R. and Tosti-Kharas J. , Calling: The Development of a Scale Measure, Personnel Psychology. (2011) 64, no. 4, 1001–1049, 10.1111/j.1744-6570.2011.01234.x.

[24] Pei Y. J. and Zhao S. M. , Study on Knowledge Workers’ Calling, Career Commitment and Job Attitudes, Journal of Management Sciences. (2015) 28, no. 2, 103–114, (In Chinese) 10.3969/j.issn.1672-0334.2015.02.010.

[25] Zhai Q. H. et al., Study on the Mediating Effect of Career Calling Between Nurses’ Job Esteem and Job Satisfaction, The Journal of Nursing Administration. (2025) 25, no. 7, 640–644, (In Chinese) 10.3969/j.issn.1671-315x.2025.07.017.

[26] Schaufeli W. B. , Salanova M. , González-romá V. , and Bakker A. B. , The Measurement of Engagement and Burnout: A Two Sample Confirmatory Factor Analytic Approach, Journal of Happiness Studies. (2002) 3, no. 1, 71–92, 10.1023/a:1015630930326.

[27] Zhang Y. W. and Gan Y. Q. , The Chinese Version of Utrecht Work Engagement Scale: An Examination of Reliability and Validity, Chinese Journal of Clinical Psychology. (2005) 13, no. 3, 268–270, (In Chinese) 10.3969/j.issn.1005-3611.2005.03.005.

[28] Zhang F. , Zhang J. , Chen Z. L. et al., Effects of a Gratitude Intervention on Job Engagement Among Newly Recruited Nurses: A Randomized Clinical Trial, Work. (2025) 80, no. 3, 1191–1201, 10.1177/10519815241289340. 40297871

[29] Podsakoff P. M. , MacKenzie S. B. , Lee J. Y. , and Podsakoff N. P. , Common Method Biases in Behavioral Research: A Critical Review of the Literature and Recommended Remedies, Journal of Applied Psychology. (2003) 88, no. 5, 879–903, 10.1037/0021-9010.88.5.879. 14516251

[30] Richardson H. A. , Simmering M. J. , and Sturman M. C. , A Tale of Three Perspectives: Examining Post Hoc Statistical Techniques for Detection and Correction of Common Method Variance, Organizational Research Methods. (2009) 12, no. 4, 762–800, 10.1177/1094428109332834.

[31] MacKinnon D. P. , Lockwood C. M. , Hoffman J. M. , West S. G. , and Sheets V. , A Comparison of Methods to Test Mediation and Other Intervening Variable Effects, Psychological Methods. (2002) 7, no. 1, 83–104, 10.1037/1082-989x.7.1.83. 11928892 PMC2819363

[32] Cheung G. W. and Rensvold R. B. , Evaluating Goodness-of-Fit Indexes for Testing Measurement Invariance, Structural Equation Modeling. (2002) 9, no. 2, 233–255, 10.1207/S15328007SEM0902_5.

[33] Geng X. , The Analysis of Mediating Effect of self-aging Sensation on Job Burnout and Job Engagement Among Nurses in Emergency Department, The Journal of Nursing Administration. (2024) 24, no. 11, 1002–1006, (In Chinese) 10.3969/j.issn.1671-315x.2024.11.016.

[34] Cai Y. , Li Q. , Cao T. , and Wan Q. , Nurses’ Work Engagement: The Influences of Ambidextrous Leadership, Clinical Nurse Leadership and Workload, Journal of Advanced Nursing. (2023) 79, no. 3, 1152–1161, 10.1111/jan.15086. 34723406

[35] Li M. T. , The Mediating Effect of Job Burnout Between Work-Family Conflict and Work Engagement Among Medical Staff, The Journal of Nursing Administration. (2025) 25, no. 3, 267–271, (In Chinese) 10.3969/j.issn.1671-315x.2025.03.016.

[36] Luo H. and Qing W. , Analysis on Current Situation and Influencing Factors of Work Engagement of Clinical Nurses in 10 Tertiary Public Hospitals in Sichuan Province, Occupation and Health. (2025) 41, no. 21, 2944–2950, (In Chinese) 10.3969/j.issn.1004-1257.2025.21.013.

[37] Chang F. F. , Wu X. B. , and Huang Y. J. , The Mediating Effects of Psychological Capital and Organizational Commitment on the Relationships Between Calling and Work Engagement Among Clinical Nurses, Chinese Journal of Nursing Education. (2023) 20, no. 12, 1475–1480, (In Chinese) 10.3761/j.issn.1672-9234.2023.12.012.

[38] Shimazu A. and Schaufeli W. B. , Work Engagement: An Emerging Concept in Occupational Health Psychology, Biosci Trends. (2008) 2, no. 1. 20103890

[39] Emerson C. , Calling to Nursing: Concept Analysis, Advances in Nursing Science. (2017) 40, no. 4, 384–394, 10.1097/ans.0000000000000185. 28990965

[40] Zhu W. , Liao H. , and Wu Q. , Organizational Silence and Work Engagement Among Chinese Clinical Nurses: The Mediating Role of Career Calling, Journal of Nursing Management. (2025) 2025, no. 1, 10.1155/jonm/7522633. PMC12181662 40547051

[41] Wang B. J. , Mediating Effect of Personal-Organizational Fit in Clinical Nurses’ Workplace Social Capital and Career Calling, The Journal of Nursing Administration. (2024) 24, no. 03, 211–215, (In Chinese) 10.3969/j.issn.1671-315x.2024.03.006.

[42] Min G. , Wang A. Q. , and Tang T. , The Mediating Effect of Career Calling on the Relationship Between Perceived Decent Work and Work Engagement Among Elderly Care Workers, Journal of Nursing. (2024) 31, no. 1, 69–73, (In Chinese) 10.16460/j.issn1008-9969.2024.01.069.

[43] Hussain A. , Xia X. , Jameel A. , Sadiq S. , Kanwel S. , and Saleem S. , Integrating Support, Trust, and Self-Efficacy to Enhance Work Engagement: the Moderating Role of Basic Psychological Needs Among Nurses, BMC Psychology. (2026) 14, no. 1, 10.1186/s40359-026-04189-y. PMC13011525 41699736

[44] Xu D. , Zhang N. , Bu X. , and He J. , The Effect of Perceived Organizational Support on the Work Engagement of Chinese Nurses During the COVID-19: The Mediating Role of Psychological Safety, Psychology Health & Medicine. (2022) 27, no. 2, 481–487, 10.1080/13548506.2021.1946107. 34190654

[45] Fuller C. M. , Simmering M. J. , Atinc G. , Atinc Y. , and Babin B. J. , Common Methods Variance Detection in Business Research, Journal of Business Research. (2016) 69, no. 8, 3192–3198, 10.1016/j.jbusres.2015.12.008.

[46] Howard M. C. , Boudreaux M. , and Oglesby M. , Can Harman’s Single-Factor Test Reliably Distinguish Between Research Designs? Not in Published Management Studies, European Journal of Work & Organizational Psychology. (2024) 33, no. 6, 790–804, 10.1080/1359432X.2024.2393462.

